# Spatiotemporal Analysis of Gastrointestinal Tumor (GI) with Kernel Density Estimation (KDE) Based on Heterogeneous Background

**DOI:** 10.3390/ijerph19137751

**Published:** 2022-06-24

**Authors:** Zhenjie Yang, Sanwei He, Huiyuan Zhang, Meifang Li, Yuqing Liang

**Affiliations:** 1 Faculty of Humanities and Social Sciences, Macao Polytechnic University, Macao, China; zjyang@mpu.edu.mo; 2 School of Public Administration, Zhongnan University of Economics and Law, Wuhan 430073, China; jsj2340@126.com; 3 Department of Geography, Dartmouth College, Hanover, NH 03755, USA; limfjx@foxmail.com; 4 School of Government, Shenzhen University, Shenzhen 518060, China

**Keywords:** heterogeneous background, gastrointestinal tumor (GI), spatiotemporal distribution, Monte Carlo simulation

## Abstract

The purpose of this study is to explore hotspots or clusters of gastrointestinal tumors (GI) and their spatiotemporal distribution characteristics and the changes over time in 293 villages and communities in Jianze County, central China, through the kernel density estimation (KDE) method based on the rarely considered heterogeneous background. The main findings were: (1) Heterogeneous background impact: there were substantial differences in the GI case rate among people of different ages and genders in Jianze County. Specifically, the GI case rate was significantly higher in the elderly population over 65 than in the population under 65, and higher in men than in women. (2) GI in Jianze County exhibited spatial specific and aggregated hotspots. The high-value spatial clusters were mainly located in Hujindian Town in the northern county, Wupu Town and Geputan Town in the middle, and Xiaxindian Town in the south. Some villages had persistent hot spots for multiple years. (3) Most GI hotspots in Jianze County were concentrated in areas with both high density of local chemical plants and with water systems in the neighbourhood. We expect that this study provides a scientific basis for exploring unknown risk factors of tumor occurrence from a spatial perspective in the future.

## 1. Introduction

Cancer is a major global public health concern that has risen to become the second cause of death due to disease worldwide after cardiovascular diseases [1]. From 2007 to 2017, the growth rate of global cancer cases was as high as 33% with an increasing number of new cancer cases in almost all countries every year. According to statistics from the International Agency for Research on Cancer (IARC), 19.29 million new cancer cases and 9.96 million cancer deaths were newly added globally in 2020 [2], and 28.4 million were expected in 2040. The cancer case and mortality rate in China have been at the medium-high level among 185 countries or regions around the world. Cancer has become the leading cause of death in China [3,4], and 36.4% of cancer deaths in China are gastrointestinal tumors [5]. Gastrointestinal cancer (GI) as a type of malignant digestive tract tumor with poor prognosis has threatened general public health in China.

Earlier research on health care mainly adopts traditional non-spatial statistic methods, such as time series analysis, descriptive statistical analysis, etc, which fails to consider the spatial factor in disease distribution and pattern. With the development of geographic information science and technology, the spatial statistical analysis of health data can provide clues in determining high-risk areas and etiological hypotheses [6]. Disease mapping is an important field in Spatial Epidemiology [7]. It can visualize the distribution of diseases in time and space, display high-risk areas and their changes over time, and therefore provide a scientific basis and hypotheses for identifying disease-related environmental risk factors, guiding further analysis.

Disease mapping for cancer analyses the temporal and spatial distribution and hot spots of cancer cases to further scrutinize the causative factors. Based on the average annual GI death toll and population in 549 administrative villages in the county, Zhou et al. (2010) used spatial autocorrelation and spatial hot spot detection to identify GI clusters in the study area [8]. Qi et al. (2012) used Bayesian spatial smoothing (NobBS) to process the upper GI death case data in 14 counties in the Huaihe River Basin, and further identified the environmental risk factors of upper GI in the area by a geographically weighted regression model [9]. Dong et al. (2014) used local spatial autocorrelation and kernel density analysis to explore the spatial agglomeration of China’s “cancer villages” from the provincial and regional spatial scales [10]. Tian et al. (2019) identified GI settlements in Xiantao City through dynamic constrained spatial clustering analysis and general KDE analysis based on the medical records of cancer cases in Xiantao City in 2017 and pointed out that the accessibility of medical facilities was correlated with GI case with geographically weighted regression results [11]. Based on the data of female breast and cervical tumors in 30 provinces of Iran from 2004 to 2009, Raei et al. (2018) drew the tumor risk distribution map at the provincial level through the constructed bivariate spatial-temporal model of the disease [12]. Based on gastric cancer data from the Iranian Cancer Registry between 2005 and 2010, Rastaghi et al. (2019) pointed out that factors such as overweight and smoking were significantly related to the occurrence of gastric cancer through a gastric cancer risk map drawn with hierarchical Bayesian model [13]. John et al. (2020), based on the clinical data recorded by the Hong Kong Hospital Authority, identified high-risk areas of oral cancer at the district-level in Hong Kong from 2013 to 2019 through the Bayesian disease map model and guided the establishment of effective preventative measures in the local control of oral cancer [14]. Overall, spatial analysis has been applied in various studies to identify high-risk areas of tumors from existing data. Few studies, however, have fully considered the heterogeneity of the research background.

Existing disease mapping studies usually ignore the differences in background populations, assuming that the populations in different regions are homogeneous and have the same population structure, which often does not conform to objective facts. Cancer is a chronic disease sensitive to age. Generally speaking, the older the age, the higher the risk of cancer. Therefore, cancer mapping based on the assumed homogeneous background may lead to wrong conclusions [15]. Kirby and others (2017) have warned that when assuming that the background population in the study area was evenly distributed, we might underestimate local cancer risk and misidentify high-risk areas [7]. In addition, the purpose of disease mapping is to identify areas that are significantly higher than normal. The abnormally high case in this area indicates the particularity of the environment in this area and paves the way for further exploration of the relationship between pollutants in the environment and diseases. Here, the “normal situation” used for comparison requires proper definition and is usually expressed in terms of the expected case rate. Traditional methods rely too much on statistical assumptions and the construction of models. In sparsely populated areas, there may be serious instability due to the increase or decrease in the number of cases. It is time to zoom in, as an accurate assessment of the real risks in such areas has the same effect on public health [16]. Finally, limited by the availability of data, the spatial resolutions of existing research are mostly mesoscales, such as provinces, cities, counties and districts, but there is less depiction of finer-scale spatial heterogeneity, which limits the accuracy of the analysis results.

The existing literature has widely acknowledged that Geographic Information System (GIS) provides a useful tool to assess the environmental exposure. Bellander et al. (2001) [17] examined the positive relationship between lung cancer and several components of air pollution such as nitrogen oxides and sulphur dioxide. Wang et al. (2010) [18] proved that spatial stratified heterogeneity is widespread in spatial data. Thus, this paper assumes the existence of spatial stratified heterogeneity when assessing environmental exposure that would determine spatial patterns of cancer cases. A geographical detector is useful to measure spatial stratified heterogeneity and test the spatial association between two variables [19]. The employment of this newly developed technique could provide valuable insights into spatial association between cancer cases and environmental exposures. Furthermore, both space and time are fundamental in epidemiology [20]. It is significant to incorporate the temporal dimension into spatial analysis. Geographical and temporal weighted regression (GTWR) has been developed to investigate local nonstationary process in both space and time [21].

Jianze County, located in central China, has collected spatial-temporal data of cancer cases at the village scale, which is rare in China. The county’s tumor case numbers have been higher than the national average for a long time [12,13,14,15,16,17,18,19,20,21,22,23,24,25,26], and the number of GI cases accounted for about half of the total number of local tumor cases. In this study, the 2009–2013 GI data in Jianze County was used as the sample data, and Shi’s KDE method under the heterogeneous background was applied to first analyze the village-scale high fever spots of gastrointestinal cancer in the county [15], and then overlap with the published data of the county’s chemical enterprises and water systems to provide scientific hypotheses and clues for further exploring the environmental risk factors of GI in the region. At the same time, the analysis process and research framework established by this study for the application of nuclear density estimation methods under the heterogeneous background can also provide references for other tumor mapping, to improve cancer prevention and control for the construction of a healthy environment.

## 2. Overview of the Study Area and Data

### 2.1. Overview of the Study Area

Jianze County, located in central China, has nine large and small rivers in its territory, including the Fu River and Hanbei River. The plain terrain is relatively high in the north and low in the south. As of the end of 2018, Jianze County has nine towns and three townships under its jurisdiction, with a total of 270 village committees and 24 neighbourhood committees, with a land area of 605.31 square kilometres and a permanent population of 537,300. Jianze County advocates its economic development strategy as a “industrial power county”. The local industrial system has been formed with plastic packaging, salt chemical industry, new building materials, machinery and electronics and food processing industries as its core, representing 55.48% of the county’s gross product. Jianze County, as a rural cancer registry and testing site, has recorded its cancer case and death toll since 2009.

### 2.2. Associated Data and Pre-Processing

#### 2.2.1. Data

In this study, we obtained from Jianze County information on 6686 cancer cases from 2009 to 2013. We adopted the sixth census data of China in 2010 with gender-age population information at the district and county level in China as the background data. Ye et al. (2019) used multi-source remote sensing data and POI data to deconstruct the census data into 100 m ∗ 100 m raster population data [27], in which each grid cell stores the population information in the 100 m ∗ 100 m spatial unit and the total population of each village can be learned through regional grid cells’ statistics.

Local chemical industry data came from the corporate information search website (https://www.qcc.com/, accessed on 15 March 2019). We extracted data for all 71 enterprises established before 2013 and running at least until 2005, in the textile industry, chemical raw material and chemical product manufacturing, metal smelting and processing industry, rubber and plastic products industry, and other chemical industries, including establishment and operating time, type of industry, business scope, and detailed company address. We performed address matching according to the enterprise address to convert it into space vector point data. The river water system data came from the National Geographic Information Public Service Platform Tiantu. Other map data, such as village and community point data in Jianze County and administrative boundary vector data came from the National Basic Geographic Information Centre.

#### 2.2.2. Data Pre-Processing

(1) Constructing the scope and boundary of the village

The township boundary is available in the existing administrative boundary data in China, while village-scale spatial data is difficult to obtain directly. In order to make full use of the collected village-scale case information, we created a Voronoi diagram based on the 293 village geo data to determine the villages boundaries through the Voronoi Map tool in ArcMap 10.7.

(2) Calculating the expected cases in various places in Jianze County

An abnormal area in a disease chart usually refers to a hot or cold spot that is higher or lower than “normal”. Therefore, before carrying out disease mapping, it is necessary to clearly define the “normal situation” (also known as background data). These “normal situations” are caused by known conventional factors, such as age, gender and others. In epidemiology, such known factors are also called interference factors. In order to highlight the effect of unknown factors on abnormal areas, it is necessary to eliminate the influence of interfering factors with normalisation to enhance the comparability with morbidity or mortality [28]. Therefore, we apply the KDE method with heterogeneous background in this study. We first obtain the expected number of GI in Jianze County to generate background data for the upcoming disease mapping. Then we pre-process the data with consideration of the population structure of each region, such as age and gender. The specific processing steps are as follows:

Due to the obvious age clustering of GI case data, the simple grouping by every five years of age is likely to cause decimal problems. Therefore, we need to redefine the new gender-age group. We calculate the original population data of the crude case rate of gender-age group, then put the similar crude case rate together to determine the gender-age grouping for the study of GI cases in Jianze County. As a result, a total of 11 gender-age groups are determined (Male 6 groups, female 5 groups), as shown in Table 1.

Based on the determined gender-age group, we used the idea of indirect normalisation to calculate the expected case of GI at each village of Jianze County, with the county population and county-level case rate as the benchmark. The calculation formula of Expect Cases for GI at each village is as follows:

$$Expected\_Cases_{i}=\frac{Actual\_Cases_{i}\times Population_{i}}{Expected\_Poulation_{i}}$$

$$Expected\_Poulation_{i}=\sum_{i}^{294}\sum_{j}^{11}Actual\_Cases_{ij}\times Standard\_rate_{j}$$

where, 

*i* = 1–294, representing the 294 villages in Jianze County; 

*j* = 1–11, representing the 11 gender-age groups; 

$Actual\_Cases_{i}$ is the number of cases of the *i*-village; 

$Population_{i}$ is the population of the village; 

$Expected\_Poulation_{i}$ is the expected population of the *i*-village; 

$Actual\_Cases_{ij}$ = the case number of *j*-gender-age group in the *i*-village, and

$Standard\_rate_{j}$ = the standard case rate of the *j*-gender-age group in the whole county, which can be calculated by total case number in the *j*-gender-age group divided by the total population of the *j*-gender-age group in the county.

Finally, the calculated expected case number is converted into a raster layer, which is used as the input file (the background data in the analysis process) of the heterogeneous background KDE tool (Density-based mapping) in the ArcHealth plug-in.

## 3. Method

### 3.1. Evaluation of GI Intensity Based on KDE(KDE) with Heterogeneous Background

KDE(KDE) was first proposed by Rosenblatt (1955) and Parzen (1962). It is a non-parametric test method for estimating unknown density functions. KDE is unique in that it does not require any prior knowledge about the data distribution, nor make any assumptions about the data distribution; rather, it starts from the data sample itself to study the characteristics of the data distribution. The calculation formula is:

$$f\left(x,y\right)=\frac{1}{nh^{d}}\overset{n}{\underset{i=1}{\sum }}K\left(\frac{d_{i}}{h}\right)$$

where:

f(x,y) = the density estimation at location (x,y)

*n* = the number of cases

*h* = the spatial bandwidth

*d* = the data dimension, the spatial distance between the case *i* and the location (x,y)

*K* = the density function

KDE involves two key issues: the location of the kernel centre and the bandwidth settings. Shi (2009) has scrutinized the issues [15] and developed the ArcHealth software (https://sites.dartmouth.edu/xunshi/archealth/, accessed on 20 March 2019). Shi divides the location of the kernel core into two types: geo-based and case based. In geo-based analysis, the research area is divided into a number of regular cells (raster pixels in the background data), and then takes the cell centre as the kernel core to calculate the cell density value. In case-based analysis, the case geo location is the kernel centre, and the calculation result is the density value of the cell. When the background is homogeneous, the calculation results of the above two kernel centre bases are consistent, while when the background is non-homogeneous, the KDE with case-based kernel core is closer to the real situation [15]. The bandwidth setting includes two aspects: bandwidth category and bandwidth size. The bandwidth category includes fixed bandwidth and adaptive bandwidth. Among them, fixed bandwidth means that all kernels use the same bandwidth and have the same value. Adaptive bandwidth refers to assigning a specific bandwidth value to each kernel according to certain rules (such as adjusting according to the population background data), resulting in different sizes kernels. We can have different kernel sizes by adapting the bandwidth to accommodate a prescribed case number for a certain population background. Generally, the smaller the bandwidth, the more details can be reflected. If too small, only the original point can be observed, with the overall trend being missed. The larger the bandwidth, the smaller the difference between locations, and the better the overall trend, with some local details ignored. Therefore, we need to finetune the bandwidth through constant adjustments and experiments.

This paper uses the KDE method with the heterogeneous background proposed by Shi [15], based on the heterogeneous background of expected case and recorded case numbers in each village, to calculate the continuous density distribution surface of GI in Jianze County. In order to explore the reliability and consistency of the results caused by different bandwidths, this study tested a total of four sets of fixed bandwidths and four sets of adaptive bandwidths. Among them, the fixed bandwidth tests 1 km, 3 km, 5 km and 10 km, and the adaptive bandwidth size is set to include at least one, three, five, and ten cases.

### 3.2. Judging the Significance of GI Intensity Based on the Monte Carlo Process

In order to determine whether the GI intensity in any location obtained in the previous step is really worthy of attention, to assess the significance of the intensity of each location we can use computer simulations to enumerate a large number of “normal” situations, and compare them to the actual situation, in order to estimate the probability of the actual situation. This simulation process is called Monte Carlo simulation, and its essence is a numerical simulation method that takes probability phenomena as the research object. Ideally, KDE has accurate information about the geographic location of each case point, but one of the common problems with disease data is that the addresses of many patients are only associated with certain regional units and cannot be accurately located [28]. In this study, the GI data is only accurate to the village scale, which is still insufficient to the expected case of 100 m ∗ 100 m. For this reason, Shi proposed a combination of the unrestricted and controlled Monte Carlo process (UCMC) and the restricted and controlled Monte Carlo simulation process (RCMC) to simulate the spatial distribution of individual GI cases to estimate the significance Probability(P) value [15]. The “restrict” here refers to the restriction of the village boundary during the case deconstruction, and the “control” refers to the significance probability in each village during the case deconstruction. The formula for calculating the *p* value of a location is:

$$pv\left(x,y\right)=\frac{k_{GE\left(x,y\right)}+1}{k+1}$$

where,

pv(x,y) = probability density value on location (x,y);

$k_{GE\left(x,y\right)}$ = the number of simulated cases with “normal” density value greater than or equal to the actual density value; and *k* = number of simulations.

Shi believes that the real hot spot should be those areas where the GI intensity value is high enough while the uncertainty is limited enough, which can be judged by the following formula:

$$H\left(x,y\right)=1,\;\;\;\;\;\;\;\;\;mean_{p,\left(x,y\right)}+std_{p,\left(x,y\right)}\times 2<\alpha \;H\left(x,y\right)=1$$

where, 

H(x,y) is an identifier. When it equals 1, location (x,y) is a significant hot spot.

When it equals 0, location (x,y) is not a hot spot.

$mean_{p,\left(x,y\right)}$ and $std_{p,\left(x,y\right)}$ are the mean and standard deviation of the *p* value at (x,y) respectively; and α is the threshold of statistical significance. In this study, the value of α is 0.001 and 0.005 to delineate the final significant hot spot area.

### 3.3. Exploring the Spatial Stratified Heterogeneity between GI Intensity and Causative Factors of GI Using Geographical Detector

Geographical detector can not only be used to test spatial heterogeneity of a single variable, but also to detect the possible causal relationship between two variables by testing the coupling between spatial distributions of two variables. The basic hypothesis behind this technique is that spatial distribution of the independent variable and the dependent variable should be similar if an independent variable imposes significant influence on a dependent variable [18]. By calculating and comparing the *q*-value of each single factor and the *q*-value after the superposition of two factors, geographical detector can judge whether there is an interaction between the two factors, as well as the strength, direction, linearity or nonlinearity of the interaction. The *q*-value is utilized to measure the spatial heterogeneity of the dependent variable *Y* and to what extent a factor *X* explains the spatial variation of *Y*.

$$q=1-\frac{SSW}{SST}$$

$$SSW=\sum_{i=1}^{m}N_{i}\sigma_{i}^{2}$$

$$SST=N\sigma^{2}$$

where i=1, 2, …, m is the strata of *Y* or *X*; $\sigma_{i}^{2}$ and $\sigma^{2}$ are the variances of the *Y* values in layer i and the whole region, respectively. SSW and SST refer to within sum of squares and total sum of squares, respectively. The *q*-value ranges from 0 to 1 and a larger value indicates that spatial heterogeneity of *Y* is more evident, or the explanatory power of the independent variable *X* to *Y* is stronger.

### 3.4. Exploring the Possible Mechanisms Causing GI Using GTWR

The data selected include a set of the cancer incidence rates and their determinants in Jianze county from 2009 to 2013. GTWR is an extension of the geographically weighted regression model and considers spatiotemporal non-stationarity. The dependent variable is the cancer incidence rate at different years across 293 villages. According to data availability, this paper chooses the possible geographic and socioeconomic factors as the independent variables, including distance to water system, distance to county centre, distance to town centre, distance to national roads, county-level roads, elevation, GDP, population, and distance to chemical enterprises. Thus, the GTWR model can be expressed as below:

$$Y_{i}=\beta_{0}\left(u_{i},v_{i},t_{i}\right)+\sum_{k}^{}\beta_{k}\left(u_{i},v_{i},t_{i}\right)X_{ik}+\varepsilon_{i}$$

where $\beta_{k}\left(u_{i},v_{i},t_{i}\right)$ is the coefficient for each variable *k* and each space-time location *i*; $\left(u_{i},v_{i},t_{i}\right)$ is the space-time coordinates of location *i*; $X_{ik}$ refers to the independent variables; and $\varepsilon_{i}$ is the error term.

## 4. Results

### 4.1. Basic Situation of GI in Jianze County

From 2009 to 2013, a total of 3296 GI cases occurred in Jianze County, with 457, 725, 657, 746, and 711 each year, respectively, with the corresponding case rate at 87.08, 138.15, 125.19, 142.15 and 135.48 per 100,000 people, showing a trend of fluctuating growth. In 2009, the GI case rate in Jianze County was still at a low level, but after the significant increase in 2010, although the case rate of GI from 2011 to 2013 had fluctuated, with even a slight decline in some years, the overall level has remained at relatively high.

**Figure 1:**
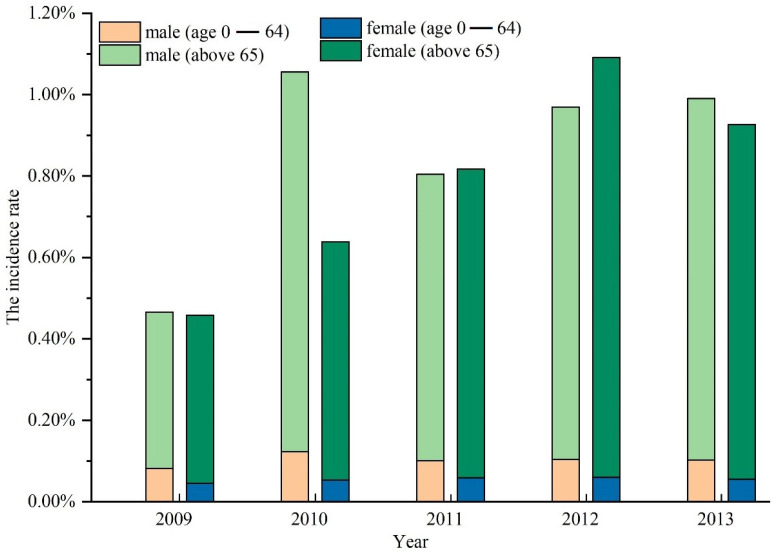
Case rate of GI in Jianzhe County by Gender-Age Group.

From the stacked histogram of the case rate of GI by gender-age group in Jianze County, as shown in Figure 1, from 2009 to 2013 significant differences exist in different gender-age groups, i.e., the case rate of GI in the elderly population over 65 years old is significantly higher than that of people under 65 years old, and the case rate of GI in men is higher than that in women. Therefore, when mapping spatial distribution of GI hot spots in Jianze County in this study, age is an interfering factor that should be excluded.

### 4.2. GI Hotspot Spatiotemporal Distribution in Jianze County

**Figure 2:**
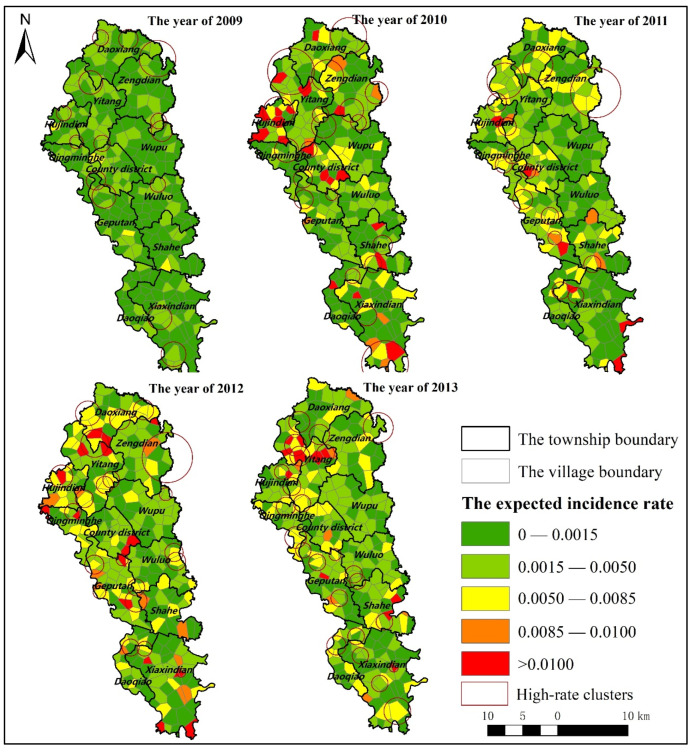
The Spatiotemporal distribution of expected case rate of GI and high-rate clusters (Authors use the software of SaTScan to detect the high-rate clusters of the expected incidence rate) in Jianzhe County (2009–2013).

Before identifying the hot spots of GI, this paper calculated the expected case rate of GI in various villages in Jianze County, and the temporal and spatial distribution is shown in Figure 2. From 2009 to 2013, the very-high and high value of expected case rate of GI in Jianze County were mostly scattered in Hujindian Town, Yitang Town, Zengdian Town, and Daodian Township in the northern part of the county, and Xiaxindian Town in the southern district; while areas with relative-high value of expected case rate of GI were scattered throughout Jianze County, and almost every township had villages belonging to areas with a relative-high expected case rate of GI. At the same time, the expected case rate of GI in Wupu Town and Wuluo Town in the central county was significantly lower than that of other towns.

**Figure 3:**
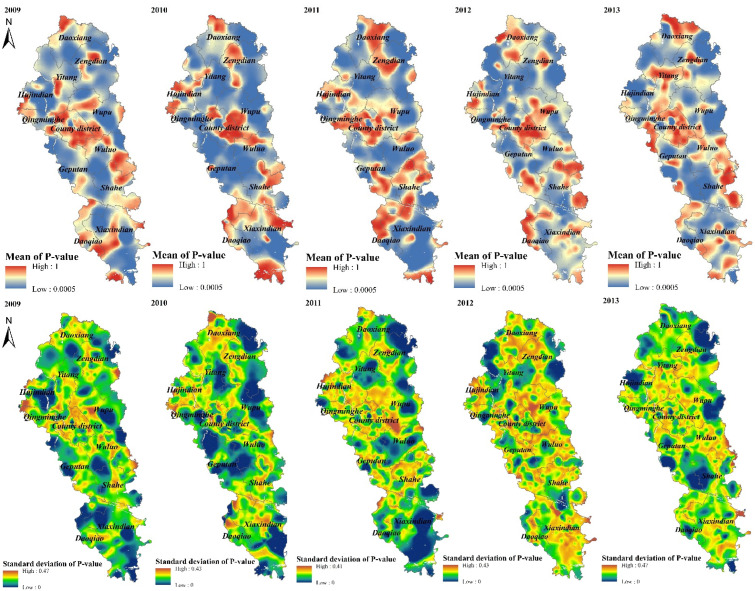
The Geo-computational Process for the hot spots of GI in Jianzhe County.

After preparing the background data for disease mapping, this article uses ArcGIS 10.7 and Archealth plug-in, developed based on the platform, to realize the KDE and Monte Carlo simulation in the heterogeneous background and then obtain the mean grid reflecting the overall stability of the density value calculated in the Monte Carlo process, and the standard deviation grid reflecting the degree of significance deviation in the Monte Carlo process. In order to make the process more comprehensive, before mapping the GI hotspot space-time distribution, this article uses the adaptive bandwidth of at least five cases as an example to show the GI hotspot geographic calculation process as in Figure 3, which includes the mean grid, standard deviation grid, and GI hotspots with a statistical significance threshold of 0.001.

Figure 3 shows that when an adaptive bandwidth of at least five cases is used, the *p* value of the standard significance deviation grid obtained by geographic calculation is coarser and the area of the high value area is larger, indicating that the obtained *p* value in the Monte Carlo process is statistically significant. When the *p* value of the mean grid of the significance is smoother and the area of the high value area is smaller, it indicates that the density value obtained in the Monte Carlo process is generally stable. In addition, in conjunction with Figure 2, it can be observed that the area of high average *p* value usually has a relatively higher expected case rate. Based on the mean and standard significance deviation grid of the *p* value, after binarizing the spatial hot spot detection results, the GI hot spot distribution under the corresponding parameters can be obtained. However, with only one set of parameters, the result is still not reliable enough to serve as the GI hot spot distribution mapping.

**Figure 4:**
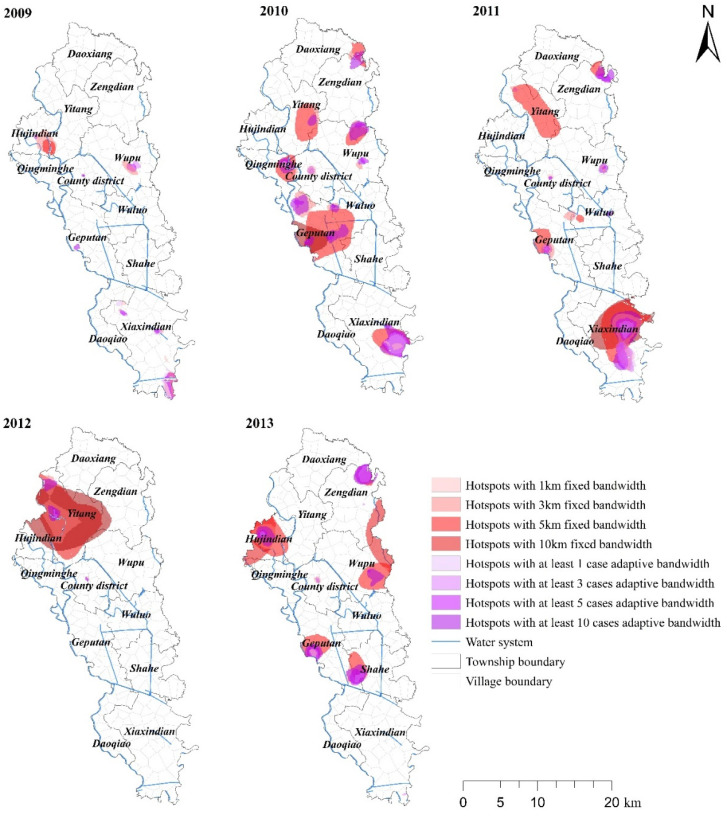
Spatiotemporal distribution of the hot spots of GI in Jianzhe County.

For reliable results, this study tests the GI hot spots with α = 0.001, and the results are shown in Figure 4. Different colours represent the hot spots formed under different bandwidths. When an area is covered by multiple colours, the area is determined as the hotspot area under multiple parameter settings, and the area can be deemed as a certain and identified GI hot spot.

Based on Figure 4, it can be seen that the spatial distribution of GI significance hot spots in Jianze County are quite different from that of the expected case rate, and the spatial patterns of GI significance hot spots in different years are also different. To be more specific:

① In 2009, when a fixed bandwidth of 1 km and an adaptive bandwidth containing at least one case were selected, the hot spots of GI first appeared only in a few villages in Xiaxindian Town in the southern county. With the gradual increase of the fixed bandwidth and the number of cases included in the adaptive bandwidth, GI hot spots also started to appear in Hujindian and Wupu towns, of the northern county. In general, with a series of bandwidths of different types and sizes, the GI hot spots in 2009 gathered in Dahe Village, Pengli Village in Xiaxindian Town, and Panzha Village in Hujindian Town, etc. In addition, due to the relatively low case rate of GI in 2009, the area of hot spots of GI generated in this year was significantly smaller than that in other years.

② In 2010, as the GI case rates increased, the area of GI hot spots in Jianze County also enlarged. With a fixed bandwidth of 1 km, the GI hotspots of Jianze County in 2010 appeared in Xiaxindian Town in the south and Jianze Town and Qingminghe Village in the middle. When the bandwidth gradually increased (3 km, 5 km), not only the area of the GI hot spots increased significantly, but it also appeared sporadically throughout the southern, central and northern areas. When the fixed bandwidth of 10 km was used, the GI hotspot area only concentrated in some villages of Geputan Town in the middle of Jianze County. When the adaptive bandwidth contains less than 10 cases, the GI hotspots mainly gathered in Sanyi Village, Xinfu Village and Taihu Village in Xiaxindian Town; when the adaptive bandwidth contains 10 cases or more, the spatial pattern of the GI hot spots was similar to that under the fixed bandwidth. In general, with a series of bandwidth conditions of different types and sizes, in 2010, the overlapping GI hot spots included Taihu Lake of Xiaxindian Town, Qiaotou Village of Geputan Town, Qingming River of Qingminghe Township, Sangang Village and Wu Dayuan Village of Pu Town, and Xudian Village of Zengdian Town.

③ In 2011, with a fixed bandwidth of 1 km, the spatial distribution of GI hot spots in Jianze County was similar to that in 2010, mainly concentrated in Aiguo Village and Taihu Village of Xiaxindian Town in the south of the county. With the gradual increase of fixed bandwidth (3 km, 5 km), the area of hot spots in Xiaxindian Town increased significantly and GI hot spots started to appear in Putan Town and Yitang Town. When the bandwidth was 10 km, the hotspot area only appeared in some villages in the north of Xiaxindian Town. When the adaptive bandwidth with at least one or three cases was used, the hotspot area only appeared in part of Xiaxindian Town, while when the adaptation bandwidth increased to include at least five or 10 cases, the hot spots started to appear sporadically in Geputan Town and Yitang Town. In general, with different bandwidth parameters, the overlapping hotspots included the central area of Xiaxindian Town and Qiaotou Village of Geputan Town in 2011.

④ In 2012, the spatial distribution of GI hot spots in Jianze County was significantly different from that of other years. With a fixed bandwidth of 1 km, only a very small area of hot spots appeared in Jianze County. When the fixed bandwidth increased (3 km, 5 km, 10 km), hot spots gradually formed at the junction of Hujindian Town and Yitang Town and the northern part of Yitang Town, and the hotspots area enlarged accordingly. When the adaptive bandwidth was applied, the spatial pattern of GI hot spots in Jianze County was similar to that with fixed bandwidth, but the hotspots area was relatively smaller. In general, with different bandwidth parameter settings, the most overlapped hot spots of GI in Jianze County in 2012 were mainly in Xinguang Village and Minwang Village of Yitang Town, followed by Lianghe Village and Panchen Village of Hujindian Town, and then the junction area between Hujindian Town and Yitang Town.

⑤ In 2013, the GI hotspots in Jianze County once again showed a scattered spatial pattern, but the specific hotspots clustered in different areas. Based on Figure 4, in 2013, there were no GI hot spots in Xiaxindian Town, where GI cases were frequently collected before, but Qiaotou Village of Geputan Town remained to be an area where multiple GI hotspots were clustered. In addition, Dayuan Village of Wupu Town, Xudian Village of Zengdian Town, Li’an Village and Sanzhao Village of Hujindian Town, and most areas at the junction of the western Shahe Township and Geputan Town also gathered many GI hot spots.

In general, except for 2012, the GI hotspots in Jianze County exhibited a scattered spatial pattern. Although the GI hotspots gathering in different areas in different years, Xiaxindian Town, Geputan Town, Hujindian Town, and Wupu Town had significant GI hot spots throughout the years.

### 4.3. Spatial Heterogeneity between Driving Factors and GI Incidence Rates in Jianze County

Since reform and opening up, China has grown from a traditional agricultural country to the world’s largest industrial manufacturing country, and the level of industrialization has achieved leapfrog development [29]. However, although the rapid advancement of industrialization has made outstanding contributions to economic development, it has also brought the already fragile ecological environment under huge industrial pollution pressure [30,31]. Environmental problems, such as air, soil and water pollution, continue to emerge [32,33], among which, the negative impact of water pollution on public health becomes a main inductor of many diseases in the country [34].

**Figure 5:**
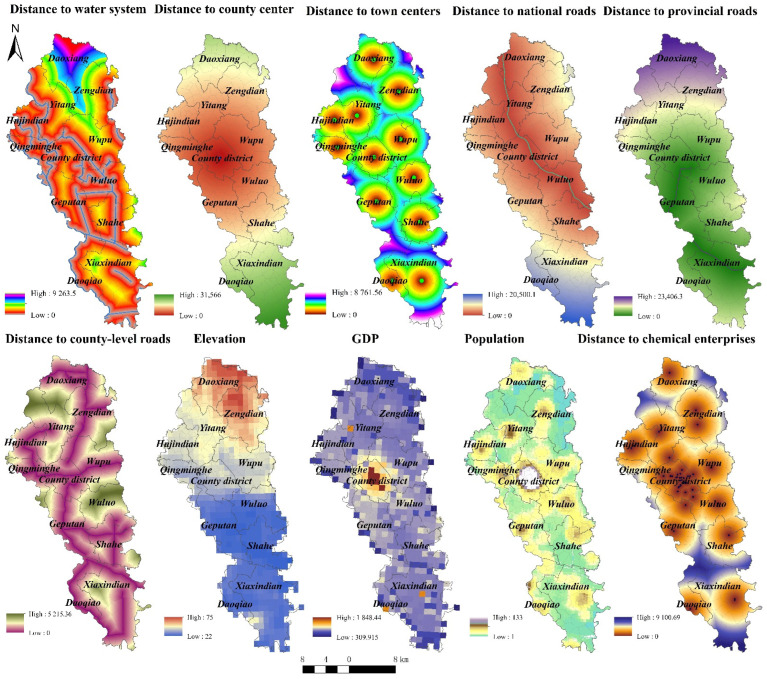
Possible driving factors of GI in Jianze County.

Spatial patterns of 10 driving factors are demonstrated in Figure 5. The overlay analysis exhibited that, although the spatial pattern of GI hot spots in Jianze County was different in each year from 2009 to 2013, most of GI Tumor hot spots were located near rivers, passing through Yitang Town, Hujindian Town and Qingminghe Township. The Fuhe River Basin had persistent GI hot spots. In other words, the spatial distribution of the GI hot spots in Jianze County was consistent with that of the water system.

Meanwhile, from the overlay analysis shown in Figure 5, it can be seen that few GI hot spots clustered here from 2009 to 2013, although the downtown of Jianze County was the most intensive area for chemical enterprises. The reason might be stricter control over production activities of chemical enterprises and environmental pollution in the downtown of the county, which mitigated the pollution of the nearby water system and resulted in almost zero GI hot spots. However, apart from the downtown, the GI hot spots in the county were not only consistent with the distribution of water systems, but also related to the spatial distribution of chemical enterprises. Specifically, in 2009, there were chemical enterprises and fine chemical plants near the GI hot spots in Wupu Town; in 2010 and 2011, the GI hot spots in Geputan Town were formed at the local industrial park, hosting chemical enterprises and material manufacturing enterprises engaged in the manufacture of chemical raw materials and chemical products, and in Xiaxindian Town, in which chemical enterprises such as phosphate chemical plants, chemical equipment manufacturers, and wall composite materials enterprises existed; in 2012, in the Fuhe River Basin, at the junction of Yitang Town and Hujindian Town, the GI hot spots gathered near the chemical enterprises, and fuel enterprises with chemical raw materials and chemical products; in 2013, in addition to the GI hot spots in Geputan Town and Wupu Town mentioned above, the GI hot spots in Hujindian Town also found a high density of chemical enterprises, with rubber powder factories and packaging materials and other chemical enterprises nearby.

Based on the above analysis, except for Jianze Township, most of the GI hotspots in Jianze County were concentrated in areas with a high density of local chemical enterprises and with water systems nearby, and the spatial distribution of the GI hotspots was consistent with that of chemical enterprises and water systems in the county. In view of this, it could be preliminarily inferred that water environment pollution caused by the county’s industrial development had directly contributed, to a certain extent, to the occurrence of GI, and the corresponding areas had therefore become GI hot spots.

Geographical detector is employed to detect spatial heterogeneity between driving factors and GI and the *q*-value is demonstrated in Table 2. There are three driving factors having significant explanatory power on GI incidence rates in Jianze: distance to water system, distance to chemical enterprises and distance to provincial roads. Existing studies have shown that water pollution is one of the influencing factors directly related to the occurrence of GI, among many external conditions. When studying the changes in spatiotemporal distribution of “cancer villages” in China, Gong and Zhang (2013) pointed out that the distribution of “cancer villages” was closely related to the distribution of rivers [35]. When studying the spatiotemporal trends of cancer villages in the country, through Pearson correlation analysis, Cui et al. (2020) found that heavy metal pollution caused by industrial wastewater discharge led to a certain extent to the formation of cancer villages [36]. Ebenstein (2012) pointed out that rapid industrialization had directly led to the deterioration of the water quality of China’s lakes and rivers. On the premise of excluding interfering factors such as air pollution, smoking rate and diet habits, his regression analysis results demonstrated that the increase in the case of GI and water quality was directly related. For each level of deterioration of water quality, the death rate for GI increased by about 9.7% [37]. Qi and others (2012) constructed a GI death risk assessment model, and observed that the surface and groundwater quality, river network density and other indicators were closely related to the increase in GI mortality [9]. Because of the shortage of water resources in rural areas of China, unpurified sewage was often used directly for farmland irrigation, which resulted in an increasing risk of local residents suffering from GI [38]. In addition, a follow-up study conducted by the Disease Control Centre also confirmed that the occurrence of GI was closely related to water pollution [39].

**Figure 6:**
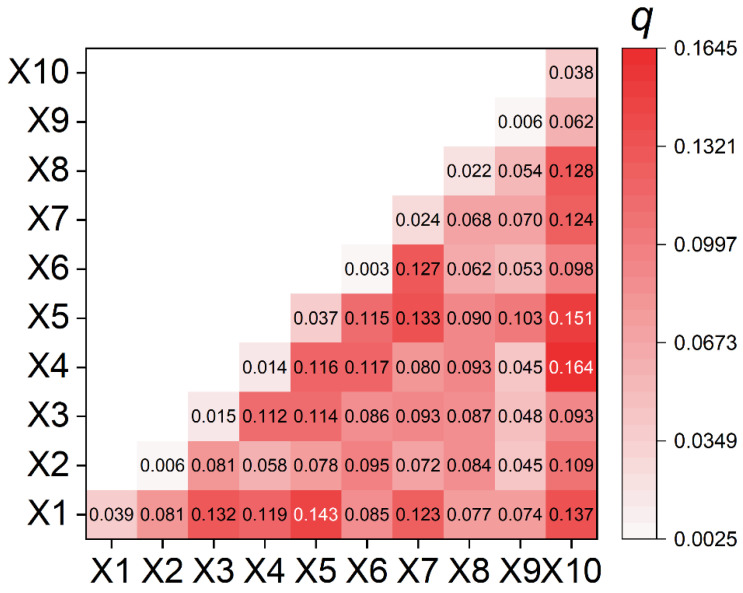
The *q*-value measured by the interaction of two factors.

In order to detect the interaction of two different driving factors, geographical detector is further used to assess whether factor X1 and X2 work together to increase or decrease the explanatory power of the dependent variable. As seen in Figure 6, the interaction of two factors is not a simple superposition process, but a nonlinear enhancement trend. This indicates that spatial differentiation of GI is not caused by a single factor, but by the interaction of different factors. Therein, the interaction of X1 and X10 (*q* is 0.164), the interaction of X5 and X10 (*q* is 0.151), and the interaction of X1 and X10 (*q* is 0.143) have better explanatory power on the spatial pattern of GI than other factors. This illustrates the dominant role of distance to chemical enterprises on the pattern of GI when examining the interaction of different factors.

### 4.4. Possible Causative Factors of GI in Jianze County Using GTWR

**Figure 7:**
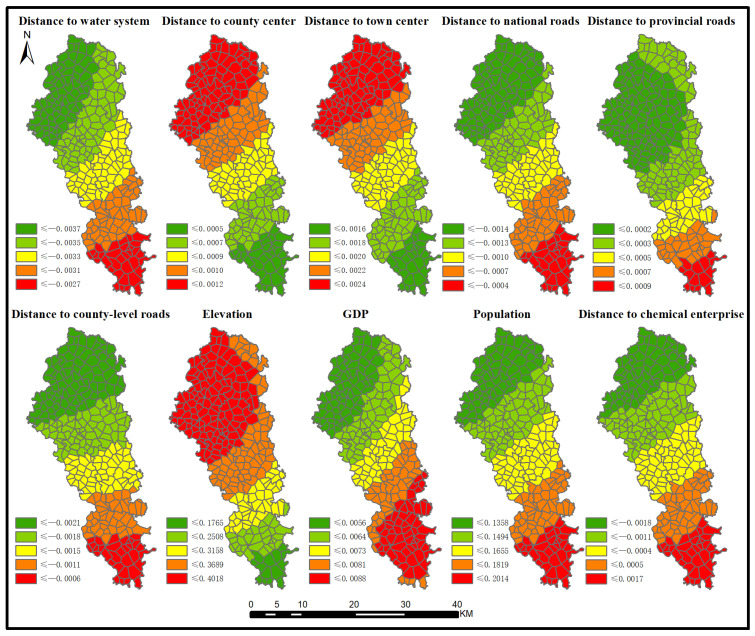
The coefficients of each driving factor when *p* is smaller than 0.01.

The GTWR model is employed to investigate the possible causative factors of GI in Jianze county by considering spatiotemporal non-stationarity. Figure 7 demonstrates the coefficients of each driving factor when *p* value is smaller than 0.01. We can see that the significant influence of each driving factor on the pattern of GI has evident spatial variation. First, the coefficient distributions of distance to water system, distance to national roads, distance to county-level roads and distance to chemical enterprises are similar in space and these driving factors have negative coefficients. High coefficients can be found in the southern part of Jianze county and low coefficients are mainly distributed in the northern part. Second, the coefficients of distance to county centre, distance to town centre and elevation are positive, denoting their positive influence on the pattern of GI. This indicates that the risk areas of GI are mostly distributed in locations at high altitudes with a remote distance to county centre and town centre. The coefficient distribution of these three factors demonstrates the pattern of high values in the north and low values in the south. Third, population and GDP have positive influence on GI, where increased production and consumption causes increased GI incidence rates. China is undergoing rapid urbanization and industrialization. In this background, Jianze county is devoted to developing its economy. Population and economic growth have significant impacts on environmental degradation.

As the main culprit in water environment pollution, industrial wastewater has the characteristics of large discharge volume, wide pollution area, and complex composition, which is extremely destructive to the water environment [40]. Because large scale township and village enterprises are characterized by high energy consumption, outdated equipment and technology [41], and environmental law is not enforced in rural areas as much as in urban areas, the water pollution caused by industrial development in rural areas is often more serious than that in urban areas. What is worse, the amount of industrial wastewater and other pollutants produced by township and village enterprises related to the papermaking, building materials, chemical and other industries also increases dramatically with the increase in output, which will further affect the public health of local residents [42]. As a county-level administrative unit that implements the “industrial county” development strategy and takes the chemical industry as the core of its economic development, Jianze County promotes the development of local industries to achieve economic growth, but the water system in the county is unavoidably affected by industrial pollution.

## 5. Conclusions and Discussion

This paper is based on a heterogeneous background study, with the application of ArcGIS 10.7 and the ArcHealth plug-in to realize the geographic calculation process composed of KDE with heterogeneous background, Monte Carlo simulation and other processes, and therefore to explore the spatial distribution of GI hot spots in Jianze County. The results show: (1) From 2009 to 2013, the GI case rate in the county exhibited a fluctuating growth trend. The distribution of hotspots had significant temporal and spatial variation. However, some villages, located in different directions such as Hujindian Town in the north, Wupu Town in the middle, Geputan Town and Xiaxindian Town in the south, have constantly appeared as GI hot spots, which are the high-risk areas for GI in the county. (2) The distribution of the water system and the kernel density of chemical enterprises in Jianze County were superimposed on the GI hotspots, and it was also found that the GI hotspots of other townships were consistent with the distribution of the kernel density and water distribution of local chemical enterprises, except for the downtown areas of the county. With a high degree of spatial consistency, it is preliminarily inferred that the high case rate of GI in Jianze County is closely related to water pollution caused by local industrial development.

External risk factors are also important incentives for GI, yet many related external risk factors are not known. The application of advanced spatial analysis methods to explore the spatiotemporal distribution of GI can help provide a scientific basis for exploring unknown risk factors from a spatial perspective. We think that the results of our study can help improve tumor prevention and control, promoting the construction of a healthy environment, to enhance the national health level and facilitate sustainable social development.

The spatial analysis method used in this paper can not only prevent disease hot spots from becoming a simple reflection of regional population density, but also reduce dependence on statistical assumptions. Full application of existing demographic information and spatial information in tumor case data accurately reveals the spatial distribution of GI hot spots in Jianze County, thus providing a solid and reliable clue for further exploration of the external risk factors of GI. However, further research is needed in the following aspects: first, in the description of the spatial distribution of GI hot spots, only one spatial measurement was applied, while multiple spatial measurements can be explored to compare the differentiation of GI hotspots in order to make the results more solid and reliable; second, after obtaining the gastrointestinal tumor hotspots in Jianze County, only qualitative overlap analysis with selective environmental elements was performed without combining quantitative analysis to further establish the correlation between external environmental factors and GI hot spots.

## Figures and Tables

**Table 1:** GI Case among age groups in Jianzhe County.

Male				Female			
Age	Cases Number	Population	Crude Case Rate	Age	Cases Number	Population	Crude Case Rate
0–34	56	132,002	42.42	0–44	126	157,764	79.87
35–44	221	50,022	441.81	45–54	183	38,891	470.55
45–54	365	39,633	920.95	55–64	310	29,847	1038.63
55–64	648	30,662	2113.37	65–79	435	20,262	2146.88
65–74	550	15,604	3524.74	≥80	115	3547	3242.18
≥75	287	6565	4371.67				

Note: The number of cases in the table refers to all GI cases in Jianze County from 2009 to 2013. The population is based on the sixth census in 2010, and the unit of case is per 100,000 population.

**Table 2:** The *q* statistics using geographical detector to examine spatial associations between driving factors and GI.

Variables	Abbr.	*q*	*p* Value
Distance to water system	X1	0.039	0.046
Distance to county center	X2	0.006	0.809
Distance to town center	X3	0.015	0.402
Distance to national roads	X4	0.014	0.426
Distance to provincial roads	X5	0.037	0.049
Distance to county-level roads	X6	0.003	0.941
Elevation	X7	0.024	0.181
GDP	X8	0.022	0.332
Population	X9	0.006	0.896
Distance to chemical enterprises	X10	0.038	0.042

## Data Availability

Publicly available datasets were analyzed in this study. This data can be found here: [https://www.qcc.com/].
